# Metformin Prevents Cisplatin-Induced Cognitive Impairment and Brain Damage in Mice

**DOI:** 10.1371/journal.pone.0151890

**Published:** 2016-03-28

**Authors:** Wenjun Zhou, Annemieke Kavelaars, Cobi J. Heijnen

**Affiliations:** Department of Symptom Research, UT MD Anderson Cancer Center, Houston, Texas, United States of America; University of Florida, UNITED STATES

## Abstract

**Rationale:**

Chemotherapy-induced cognitive impairment, also known as ‘chemobrain’, is now widely recognized as a frequent adverse side effect of cancer treatment that often persists into survivorship. There are no drugs available to prevent or treat chemotherapy-induced cognitive deficits. The aim of this study was to establish a mouse model of cisplatin-induced cognitive deficits and to determine the potential preventive effects of the anti-diabetic drug metformin.

**Results:**

Treatment of C57/BL6J mice with cisplatin (cumulative dose 34.5mg/kg) impaired performance in the novel object and place recognition task as well as in the social discrimination task indicating cognitive deficits. Co-administration of metformin prevented these cisplatin-induced cognitive impairments. At the structural level, we demonstrate that cisplatin reduces coherency of white matter fibers in the cingulate cortex. Moreover, the number of dendritic spines and neuronal arborizations as quantified on Golgi-stained brains was reduced after cisplatin treatment. Co-administration of metformin prevented all of these structural abnormalities in cisplatin-treated mice. In contrast to what has been reported in other models of chemobrain, we do not have evidence for persistent microglial or astrocyte activation in the brains of cisplatin-treated mice. Finally, we show that co-administration of metformin also protects against cisplatin-induced peripheral neuropathy.

**Conclusion:**

In summary, we show here for the first time that treatment of mice with cisplatin induces cognitive deficits that are associated with structural abnormalities in the brain. Moreover, we present the first evidence that the widely used and safe anti-diabetic drug metformin protects against these deleterious effects of cancer treatment. In view of the ongoing clinical trials to examine the potential efficacy of metformin as add-on therapy in patients treated for cancer, these findings should allow rapid clinical translation.

## Introduction

Thanks to the increased efficacy of cancer therapy there are now nearly 14 million cancer survivors in the United States alone[1]. However, many suffer from the long-term side effects of cancer treatment. Chemotherapy-induced cognitive impairment, also known as *chemobrain*, is a common negative side effect of cancer treatment that is also frequently reported by patients treated for tumors outside the central nervous system. These cognitive deficits often last long into survivorship and negatively impact quality of life[2–7]. Chemotherapy-induced cognitive impairment was noted in 78% of the cross sectional and 69% of prospective longitudinal studies performed between 1995 and 2012 in patients treated for breast cancer[4]. Formal neuropsychological testing reveals decreases in processing speed, memory, executive functioning and attention[2]. Advanced neuroimaging techniques show structural alterations in white and gray matter as well as specific regional changes in brain activity and more global disruptions of connectivity in patients suffering from chemotherapy-induced cognitive impairments[8–11]. Most studies on chemobrain have focused on breast cancer patients[12–14]. Preclinical studies in rodents have shown that the combination of drugs commonly used for breast cancer (e.g. methotrexate, 5-Fluorouracil and cyclophosphamide) induce cognitive impairment that is associated with neuro-inflammation and impaired neurogenesis[15–17]. There is emerging evidence that cognitive impairments also frequently develop in cancer patients treated with platinum-based agents[18]. Platinum-based compounds, such as cisplatin, are part of standard treatment for numerous malignancies including head and neck, testicular, gynecologic and non-small cell lung cancer[19–24]. Cisplatin penetrates into the brain where it inhibits neuronal stem cell proliferation[25]. There are a few studies indicating that cisplatin treatment reduces cognitive function in juvenile rats and adult mice[26–29]. However, these earlier studies did not examine the effects of cisplatin on brain structure.

In a previous study, we showed that cisplatin-induced peripheral neuropathy could be prevented by co-administration of the anti-diabetices drug metformin[30]. metformin crosses the blood brain barrier [31] and has neuroprotective effects in models of ischemic stroke [24, 26] and inflammation-induced brain damage [31]. There is also evidence that metformin promotes the differentiation of microglia towards an M2 suppressive/wound healing phenotype that could contribute to metformin’s beneficial effects on brain damage[31].

Metformin is widely used for treatment of type 2 diabetes, is well tolerated, and safe. In cancer patients, there is evidence mainly from retrospective studies that metformin may prevent recurrence and to enhance the effect of cancer treatment[25, 32].

The first aim of this study was to establish a mouse model of cisplatin-induced cognitive impairment and identify the underlying structural cerebral abnormalities. The second aim was to determine whether treatment with metformin protects against these cisplatin-induced functional and structural deficits.

## Materials and Methods

### Animals

Fifty-six female C57/BL6J mice (8–10 weeks old, The Jackson Laboratory, Bar Harbor, ME) were individually housed on a 12h light/dark cycle (lights on 6:00 am). Water and food were available *ad libitum*. All behavioral tests were performed during the light phase of the cycle. The study was conducted in accordance with NIH guidelines for the care and use of animals and under protocols approved by the Texas A&M Institutional Animal Care and Use Committee # 12020.

### Drug Administration

Mice were intraperitoneally (i.p.) treated with cis-diamineplatinum(II) dichloride (cisplatin) (Sigma-Aldrich, St Louis, MO) (2.3mg/kg per day) or saline for 3 cycles consisting of 5 daily injections followed by 5 days without injections. The total cumulative dose of cisplatin was 34.5mg/kg. metformin hydrochloride (metformin) (Millipore, Solon, OH) (100mg/kg) or saline was given i.p. for seven days starting 1 day prior to the first injection of cisplatin of each cycle and including 1 day after the last dose of cisplatin in each cycle. Body weight was monitored.

### Behavioral Assays

#### Novel Object-Place Recognition Test

The novel object and place recognition test (NOPRT) was performed as described by Le Merrer et al. [33] with minor modifications. Briefly, starting after the final dose of cisplatin, mice were habituated to the test arena (25(L)×25(W)×50(H) cm) for 5min/day for 5 days. On day 6, two identical objects were placed in the arena and the mouse was permitted to freely explore the arena for 5 min. After that, the mouse was returned to its home-cage and the arena, including the two objects, was cleaned with 70% ethanol. Seven minutes later, the mouse was returned to the open arena, where one object was replaced by a novel object with different shape (but made out of the same material to avoid potential interference of deficits in sense of touch or smell) and placed in a different location and allowed to explore for another 5 min. All sessions were recorded for later analysis. Sniffing, climbing and touching the objects were regarded as the exploration behavior and exploration times of the familiar and novel object were scored manually by a trained technician blinded to treatment and were validated by a second investigator. The discrimination index was calculated as (time with novel-time with familiar object)/total exploration time of both objects. The NOPRT was performed during the light phase of the day/night cycle. Preliminary studies showed a similar effect of cisplatin on behavior in the NOPRT when the test was performed during the dark phase.

#### Social Discrimination Test

The social discrimination test according to [34] with minor modifications was performed two-three days after the novel object-place recognition. One day prior to the training and testing day, the test mice and the conspecific 6 week-old juveniles to be used in the test were isolated and moved to a new home-cage with clean bedding chips. During the training session, one juvenile protected by a mesh wired enclosure was moved into the home cage of the test mouse for 5 min. The test mouse was permitted to freely explore. Eighty minutes later, the now familiar juvenile used previously and a novel juvenile in two separate wired mesh enclosures were placed in the home cage of the test mouse for 5 mins. Behavior during the training and test phase was recorded on video and scored manually by a trained investigator blinded for treatment and were validated by a second investigator. The social discrimination index was calculated as (time with novel-time with familiar mouse)/total exploration time.

#### Von Frey Test for Mechanical Allodynia

Chemotherapy induced mechanical allodynia was measured as the hind paw withdrawal response to von Frey hair stimulation using the up-and-down method as we described previously[30,35]. Mice were placed in a plastic cage (10×10×13cm^3^) with a mesh iron floor for 30 min before testing. Subsequently, a series of von Frey hairs (0.02, 0.07, 0.16, 0.4, 0.6, 1.0 and 1.4 g) (Stoelting, Wood Dale, IL) were applied to the mid-plantar of the hind paw starting with 0.16g hair. A clear paw withdrawal, shaking or licking was regarded as a positive response. Whenever a positive response was observed, a next hair with lower g force was applied; and in the case of negative response, a next hair with higher g force was applied. Five consecutive stimuli were recorded after the first positive response as g force and 50% paw withdrawal threshold were converted using the method described previously[30,35].

#### Immunohistochemistry

Four weeks later after the last injection of cisplatin, mice were euthanized by an overdose of CO_2_. For white matter staining, mice were perfused with 4% formaldehyde/0.01M PBS (pH7.4). Fixed brains were embedded in paraffin, and coronal section (10μm, -1.58 mm from bregma) were stained with mouse anti-myelin basic protein (MBP) antibody (1:2000) (Sternberger Monoclonals, Dedham, MA) followed by secondary biotin labeled antibody (1:400) (Vector Lab., Burlingame, CA) and developed with ABC reagent (Vectastain, Vector Lab.m, Burlinggame, CA). We captured images of the cingulate cortex and selected four continuous areas to cover the larger part of cingulate gyrus in each image. The coherence of the myelin staining in the cingulate gyrus region was quantified using Fiji-Image J software as we described previously[36]. Coherency of the staining was quantified using the tool in Image J. Final data were expressed as average of coherency of each mouse. Glial stainings were performed on perfusion-fixed frozen brains were frozen as previously described[35]. Coronal sections (6–7μm) were labeled with rat-anti-mouse CD11b (1:300) (BD Biosciences, San Jose, CA) or mouse-anti-mouse GFAP (1:200) (Acris Antibodies, San Diego, CA) antibodies followed by secondary fluorescent antibodies (1:1000) (Alexa Fluor, Eugene, OR). As a negative control primary antibody was omitted. Images were captured with a Leica confocal microscope (Leica, CTR4000) and analyzed with Image J.

### Golgi Staining

After CO2 asphyxiation and decapitation, mouse brains were quickly removed and flushed with ice-cold Milli-Q water twice. Brains were stained using the FD Rapid GolgiStain kit (FD NeuroTechnologies, Columbia, MD) following manufacturer’s protocol. After staining, brains were quickly frozen and 50μm sections were cut at -1.58 mm from bregma using a Leica cryostat (CM3050S). Two representative pyramidal neurons in the cingulate cortex from each mouse were selected by an investigator blinded to group for detailed analysis.

For quantifying dendritic spines, Fiji-ImageJ was used to skeletonize the dendrites on neuron. When reconstructing the neurons, the dendrites from the other neighbor neurons were automatically deleted. For evaluating the morphological change of neurons after cisplatin and metformin, Neuromanic software (Neuromantic V1.7.5) was used to semi-automatically re-construct a single neuron from the original picture. Because preliminary inspection of the photographs indicated that the dendrites of pyramidal cell in the cingulate cortex showed the most prominent change after cisplatin-treatment, we focused on this area. The skeletonized neuron was analyzed by Scholl analysis using Fiji-Image J and the number of intersections versus the radius was plotted.

Spine density was quantified on the same neurons as used in the Sholl analysis. Straight dendrites, which were around 40μm away from the cell body, were analyzed. Spine density was quantified as the number of protrusion elements on dendritic branches per mm dendrite length.

### Statistical Analysis

The data are expressed as means ± SEM. Statistical analysis was performed with SPSS 21.0. We used two-way ANOVA, or two-way repeated-measures ANOVA according to the experimental design. Raw data are available in the supplementary data file (S1 File). *Post-hoc* analysis was conducted using Tukey or LSD test. All experiments were repeated 3–4 times.

## Results

### Cisplatin induces cognitive impairment that is prevented by pre-treatment with metformin prior to cisplatin

**Fig 1:**
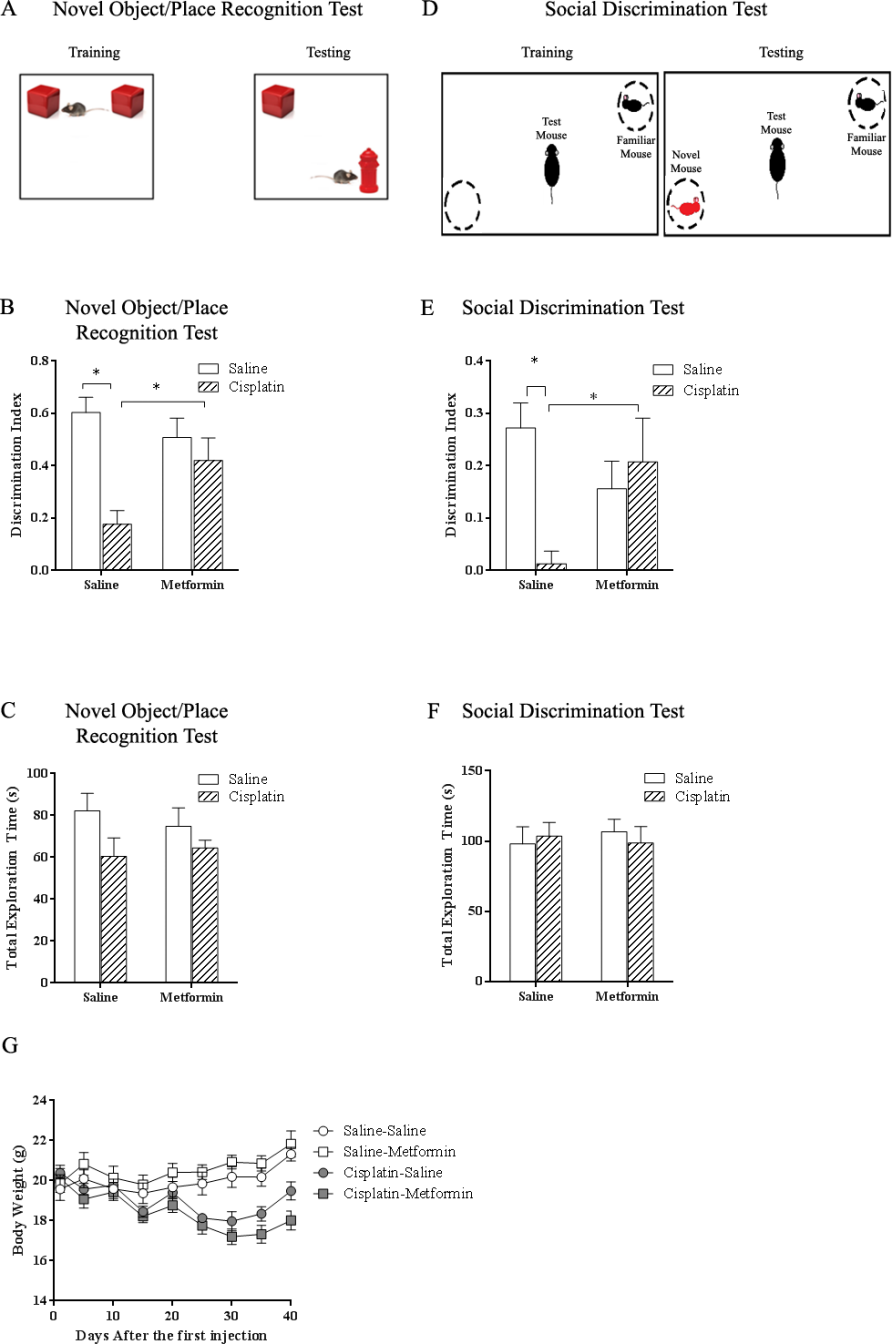
Effects of cisplatin and metformin on cognitive function. Mice received three cycles (5 daily injections of 2.3 mg/kg i.p followed by 5 days without injections) of cisplatin treatment with or without metformin (100 mg/kg i.p.). (A) Set up of novel object/place recognition test. (B) Effect of cisplatin and metformin on performance in the novel object/place recognition test. Data were analyzed by two-way ANOVA (interaction effect: Cisplatin×metformin, F(1,46) = 6.465, p<0.05) followed by LSD. (C) The total interaction time with novel and familiar object did not differ between groups. Data were analyzed by two-way ANOVA. (D) Set up of the social discrimination test. (E) Effect of cisplatin and metformin on performance in the social discrimination test. Data were analyzed by two-way ANOVA (Interaction: Cisplatin×metformin, F(1,44) = 4.42, p<0.05) followed by LSD. (F) The total interaction time during the test did not differ between groups. Data were analyzed by two-way ANOVA. (G) Effect of cisplatin and metformin on body weight (Time×Cisplatin, F(3,95) = 48, p<0.05). All data are expressed as mean±SEM. *, p<0.05. n = 10–14 per group.

Mice were treated with daily i.p. injections of cisplatin (2.3mg/kg) for 5 days followed by 5 days of rest for 3 three cycles). At 6–8 days after completion of cisplatin treatment, mice were tested in in the novel object and place recognition test (NOPRT; Fig 1A) to assess the effect of cisplatin on cognitive function. During the training phase mice were exposed to two identical objects. After 7 min. in their home cage, they were exposed to one familiar object and a novel object in a novel location. A reduction in preference for the novel object/place is indicative of cognitive impairment [37]. The data in Fig 1B show that cisplatin treatment significantly reduced performance in the NOPRT. More importantly, administration of metformin (100 mg/kg/day from one day before until one day after each cycle of cisplatin) prevented this adverse effect of cisplatin. There were no group differences in total interaction times during the test session (Fig 1C) or in time to first interaction with the novel object (S1 Fig), indicating that the results are not influenced by potential changes in overall activity or lack of motivation. In addition, cisplatin also reduced performance in a novel place recognition test, when two identical objects were used (S2 Fig). Cisplatin also reduced performance in the social recognition test, in which the preference for a novel mouse is assessed as an indication of cognitive function. (Fig 1D and 1E) Co-administration of metformin prevented the cisplatin-induced reduction in preference for the novel mouse. Total interaction times did not differ between groups as well (Fig 1F).

The cisplatin-induced reduction in body weight was not affected by co-administration of metformin. Recovery of body weight after completion of cisplatin-treatment was similar in both groups (Fig 1G).

### Metformin prevents development of cisplatin-induced mechanical allodynia

**Fig 2:**
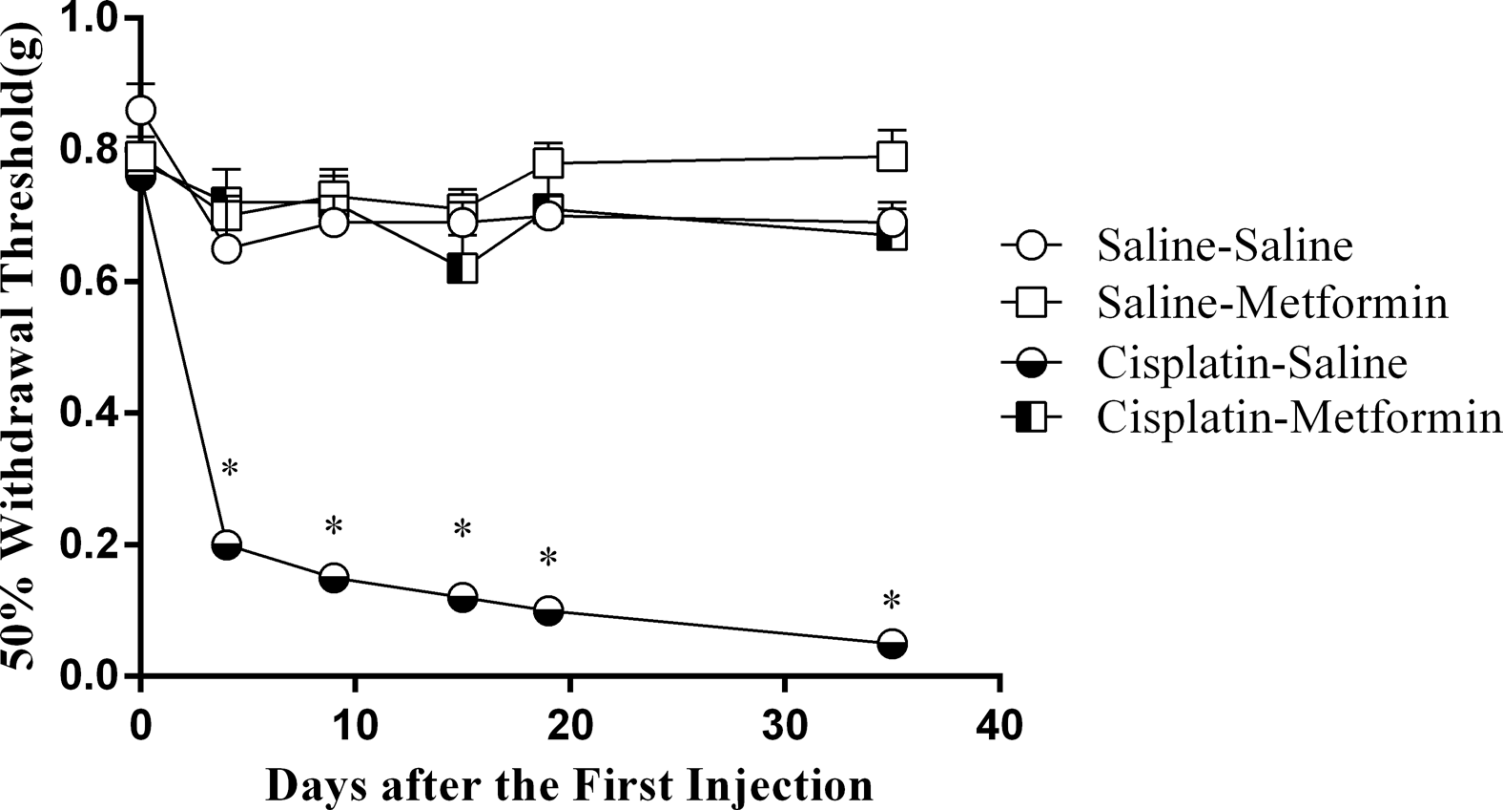
Effect of metformin on mechanical allodynia induced by cisplatin. Mice were treated with cisplatin and metformin as in Fig 1. Mechanical allodynia as an indicator of neuropathy was assessed over time using von Frey hairs. Co-administration of metformin (100mg/kg) prior to the cisplatin treatment prevented the development of mechanical allodynia. Data were analyzed by Two-way repeated measures ANOVA (Time×Cisplatin×metformin, F(4,162) = 5.78, p<0.05) followed by LSD. All data are expressed as mean±SEM. *, p<0.05. n = 10–14 per group

We previously showed that co-administration of metformin at a dose of 200 mg/kg/injection prevented the mechanical allodynia in the hind paws induced by two cycles of cisplatin[30]. The data in Fig 2 show that co-administration of a lower dose of metformin (100mg/kg) as used in the present study on cognition, also completely prevented the mechanical allodynia induced by three cycles of cisplatin. These findings indicate a robust protective effect of metformin on cisplatin-induced peripheral neuropathy.

### Effect of cisplatin and co-administration of metformin on the organization of myelin basic protein-positive fibers

**Fig 3:**
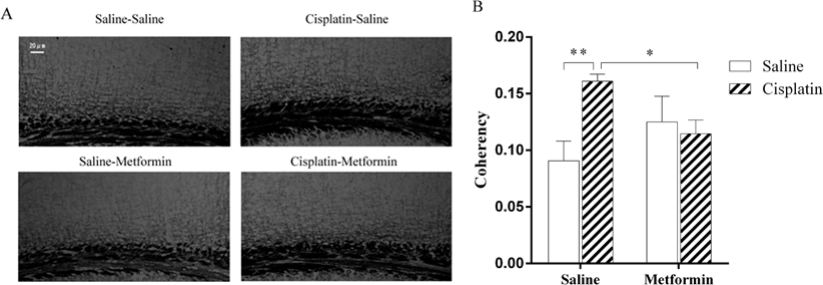
Effects of cisplatin and co-administration of metformin on the organization of myelin basic protein-positive fibers in the cingulate cortex. Mice were treated with cisplatin and metformin and brains were collected after behavioral analysis. MBP staining patterns in the cingulate cortex were analyzed as a measure of integrity of myelinated fiber networks. (A) Representative examples. (B) Pictures of all sections were captured using a Leica microscope and quantified by Fuji ImageJ with a coherency plugin. Data were analyzed by two-way ANOVA (cisplatin×metformin, F(1,15) = 17.36, p<0.01) followed by Tukey post hoc testing. Scale Bar: 20μm. All data are expressed as mean±SEM. *, p<0.05; **,p<0.01. n = 4–5 per group

Next, we analyzed myelin basic protein (MBP) staining patterns in the cingulate cortex in our mouse model of cisplatin-induced cognitive impairment as a measure of white matter integrity. This area was selected, because initial screening indicated that this was the area with the most prominent changes. In addition, there is evidence for the importance of this region in cognitive function and in particular in spatial recognition [38,39]. The data in Fig 3 show that cisplatin induced an increase in the coherency of MBP^+^ fibers in the cingulum, which is indicative of a decrease in white matter complexity. Metformin treatment, however, completely prevented the cisplatin-induced decrease in coherency of MBP^+^ fibers in the cingulum (Fig 3).

### Morphological Changes in Pyramidal Neurons in the Cingulate Cortex

**Fig 4:**
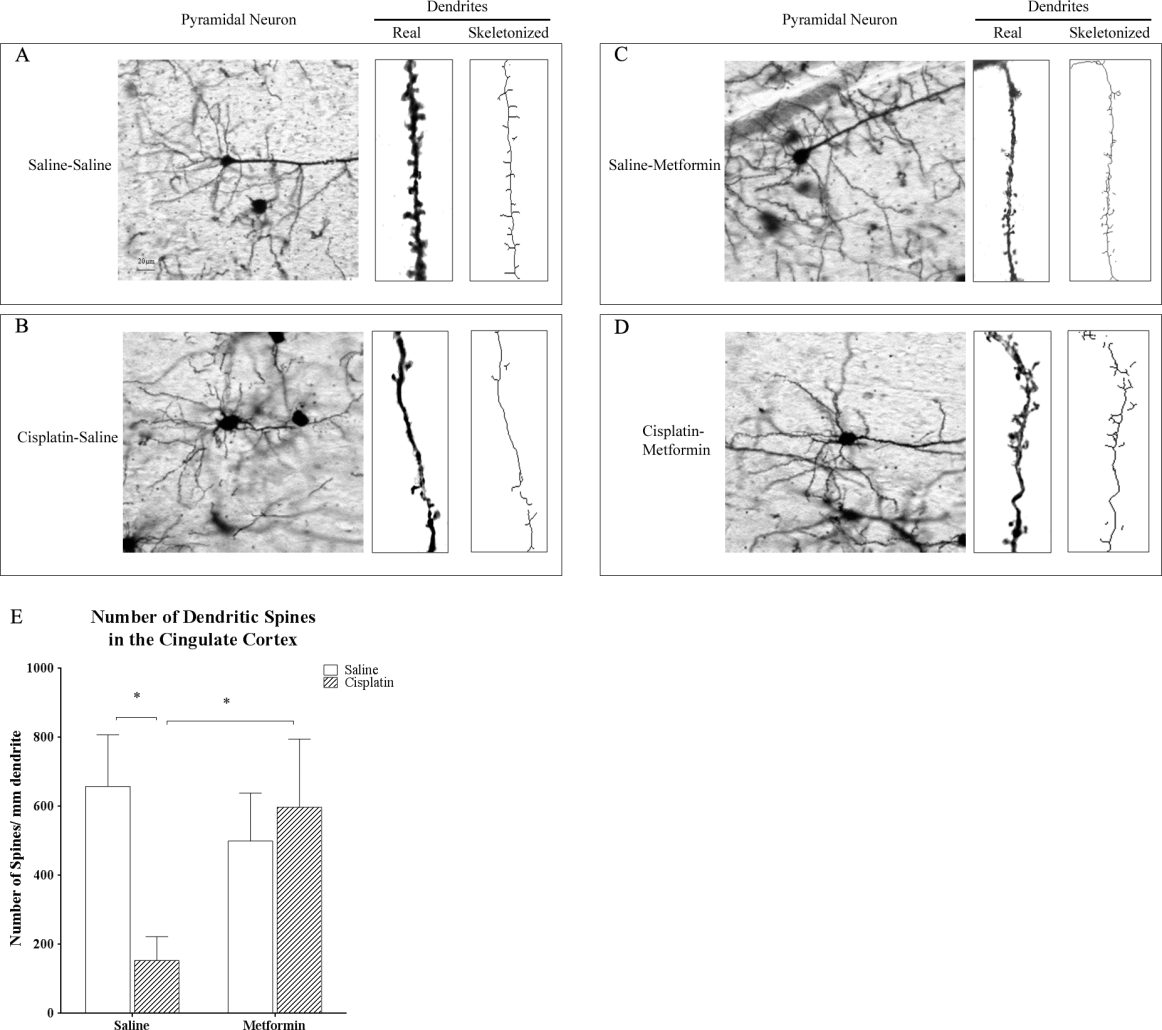
Effect of cisplatin and metformin on dendritic spine density Brains of mice treated with cisplatin and metformin were stained using a Golgi-staining kit and sliced were analyzed for dendritic spine density on pyramidal neurons in the cingulate cortex. (A-D) representative examples of actual images, an actual dendrite and a skeletonized dendrite. (E) Quantified data were analyzed by two-way ANOVA (cisplatin×metformin, F(1,17) = 22.26, p<0.05) followed by Tukey. Scale bar: 20μm. All data are expressed as mean±SEM. *, p<0.05. n = 5–6 per group

**Fig 5:**
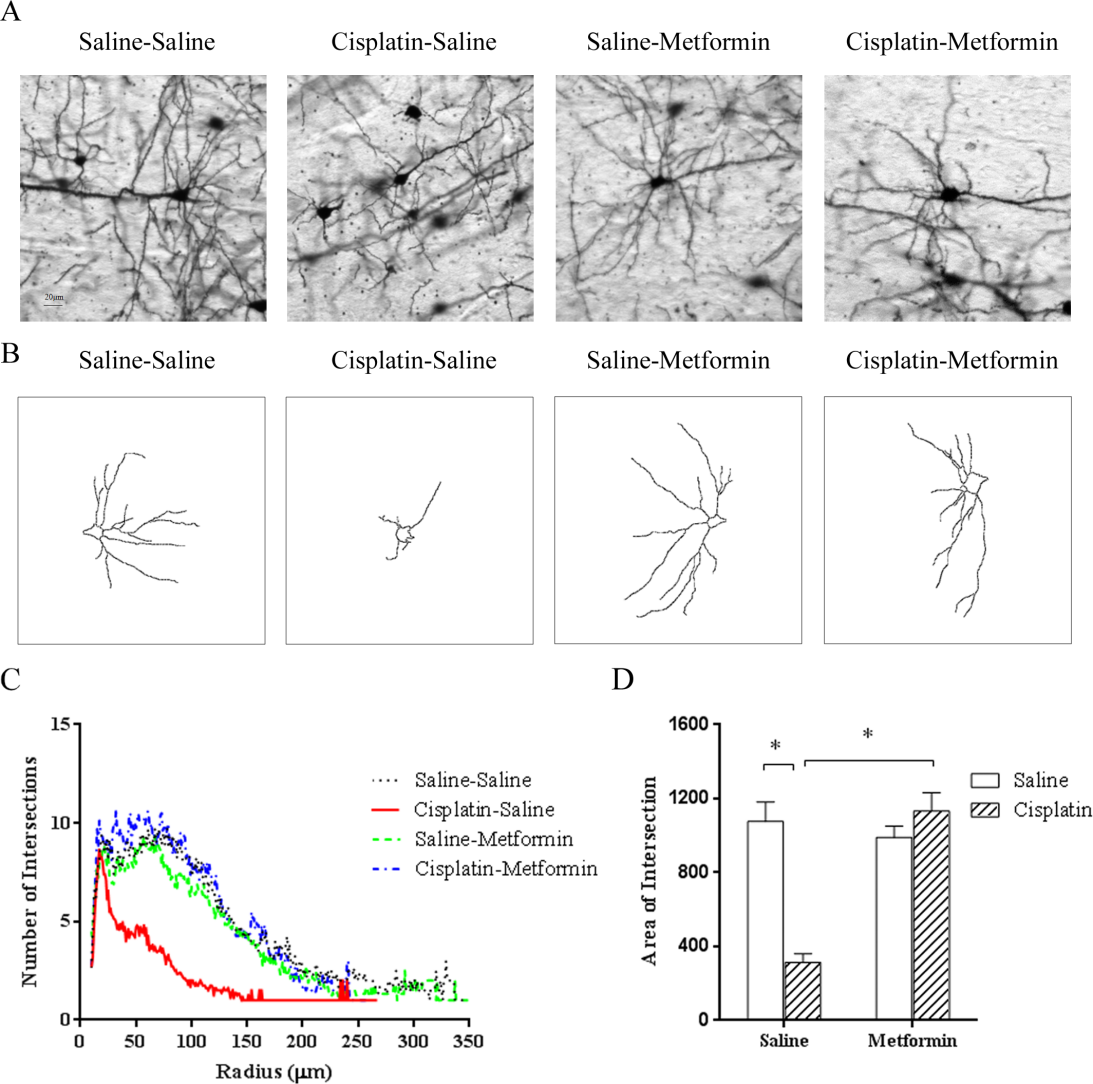
Effect of cisplatin and metformin on dendritic branching. Sholl-analysis of pyramidal cells in the cingulate cortex of Golgi-stained brains was performed. (A) Examples of actual and (B) reconstructed pyramidal cells. (C) Plot of intersection number vs radius. (D) Areas under the curve of plots in (C) were analyzed Two-way ANOVA (cisplatin×metformin, F(1,31) = 34.81, p<0.05) followed by LSD. Scale bar: 20μm. All data are expressed as mean±SEM. *, p<0.05. n = 8–11 per group.

To address the question whether cisplatin affects dendritic spines, brains obtained from mice treated with cisplatin +/- metformin were collected after completion of behavioral assessments. Brains were stained by Golgi immersion and sections were screened for morphological abnormalities. Similar to what was observed when examining MBP staining, the cingulate cortex seemed to be most affected. Therefore, we focused on this area to quantify spine density of pyramidal cells. Treatment with cisplatin dramatically reduced dendritic spine density (Fig 4). In line with the protective effect of metformin on cognitive function, co-administration of metformin prevented this cisplatin-induced decrease in spine density (Fig 4). We also analyzed the effect of cisplatin and co-administration of metformin on arborization of neurites of pyramidal cells in the brain as visualized by Golgi immersion and quantified by Sholl analysis. The results in Fig 5 demonstrate that cisplatin treatment reduced dendritic branching. Co-administration of metformin protected against the cisplatin-induced reduction in neurite arborization (Fig 5).

### Effects of cisplatin and metformin on brain glial activation

**Fig 6:**
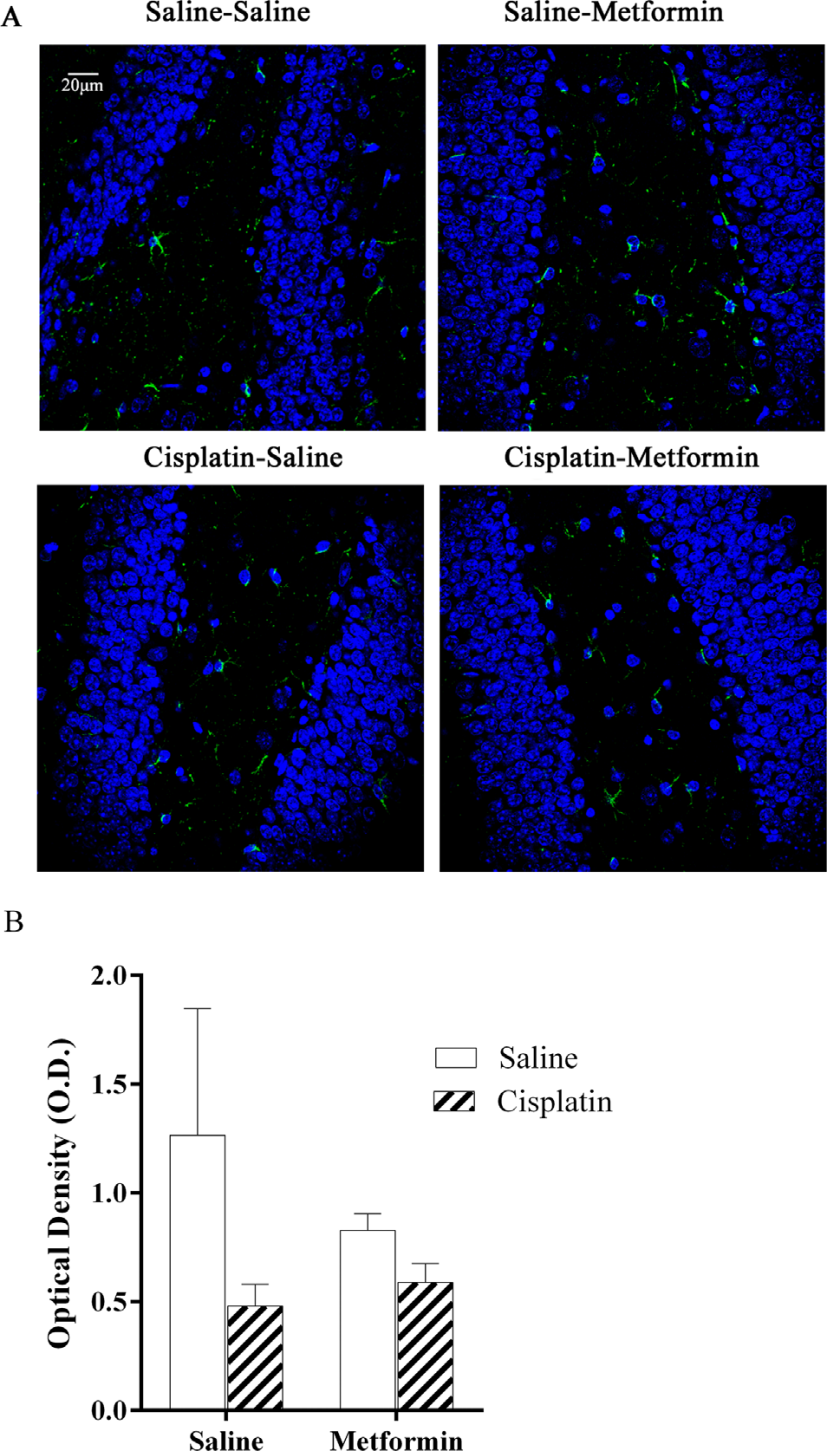
Astrocyte activation in brain of mice treated cisplatin and metformin. Expression of GFAP as a measure of astrocyte activation was analyzed in hippocampus. (A). Green: GFAP; Blue: DAPI. (B) Quantification of GFAP expression. Data were analyzed by two-way ANOVA (cisplatin×metformin, F(1,16) = 0.8265, p>0.05). Scale bar: 20um. All data are expressed as mean±SEM. *, p<0.05. n = 5–6 per group

Neuropathic pain induced by platinum-based compounds is associated with a persistent increase in the expression of GFAP and other markers of astrocyte activation in the spinal cord [40–42]. However, in the brain of cisplatin-treated mice, we did not observe increases in GFAP expression (Fig 6). In addition, we do not have evidence for microglial activation as assessed by analyzing Iba-1 expression in the brains of cisplatin-treated mice.

## Discussion

We developed a mouse model of cisplatin-induced cognitive impairment and demonstrate that the anti-diabetes drug metformin protects against the cisplatin-induced deficiencies in cognitive function. Specifically, we showed that cisplatin-treatment induced deficits in spatial orientation and memory in the NOPRT and the social discrimination task. Administration of metformin from one day before until one day after each cycle of cisplatin prevented this adverse effect. In addition, cisplatin-induced mechanical allodynia, which we used as a measure of peripheral neuropathy, was completely prevented by administration of metformin. These results indicate a robust protective effect for metformin against cisplatin-related cognitive deficits and neuropathic pain. We also show that cisplatin-treatment induced morphological abnormalities in the brain on the level of white matter organization, neuronal arborization, and dendritic spine density. In line with its efficacy against cisplatin-induced behavioral abnormalities, metformin also protected against the associated morphological abnormalities in the brain.

Patients treated for cancer with platinum-based compounds frequently develop structural abnormalities in the brain as detected by neuroimaging [8,9]. The increase in coherency of myelin basic protein (MBP)^+^ fibers that we observed in the brains of these cisplatin-treated mice may underlie the impaired white matter integrity detected by neuroimaging in patients treated with platinum-based compounds and reporting cognitive deficits[8,9]. We also detected decreases in neuronal arborization and dendritic spine density in the cingulate cortex. Such changes are known to reflect alterations in the strength and functionality of resident synapses[43–46]. It is of interest that the morphological alterations we observed, such as a decrease in dendritic spine morphology and dendritic arborization, are commonly seen in models of neurodegeneration and correlate with altered synaptic function and behavioral deficiencies[43–45]. We do not yet know how long these cisplatin-induced abnormalities remain, but our data indicate that cisplatin induces a mild cerebral neurodegeneration that might reflect increased vulnerability for advanced aging and/or degenerative diseases, such as Alzheimer’s. Importantly, prevention of the functional cognitive deficits by co-administration of metformin was associated with preservation of structural integrity, indicative of a causal relationship between preservation of structure and function.

Previous studies have shown beneficial effects of metformin in models of ischemic brain damage. For example, preventive administration of metformin before exposure to cerebral ischemia reduced brain damage in mice[47,48]. Moreover, treatment of mice with metformin starting within 24 h after stroke enhanced recovery and improved behavioral deficits[49]. In these studies, suppression of inflammatory activity and reduction of oxidative stress were proposed as mechanisms contributing to the beneficial effects of metformin in these models[47–49]. Emerging evidence suggests that cognitive impairment in patients with cancer is associated with persistent neuroinflammation[15–17]. There is evidence that neuropathic pain induced by platinum-based compounds is associated with a persistent increase in the expression of GFAP and other markers of astrocyte activation in the spinal cord [40–42], although there are also conflicting data [50]. Notably, we did not observe increases in GFAP expression or microglial activation in the brains of our cisplatin-treated mice. These findings argue against a prominent role of neuroinflammation in cisplatin-induced cognitive impairment and the protective effect of metformin.

It is well known that cisplatin induces mitochondrial abnormalities in the peripheral nervous system that are thought to underlie the development of neuropathy. In addition, we have preliminary evidence that cisplatin-induced cognitive impairment is associated with reduced mitochondrial health in cerebral synaptosomes. Metformin is known to increase mitochondrial biogenesis and respiration in various cell types[51]. Moreover, in neuronal cells, metformin has been shown to inhibit mitochondrial damage via an AMP-activated protein kinase- dependent pathway[52]. It remains to be determined whether metformin’s protective effect on cisplatin-induced cognitive impairment and peripheral neuropathy is associated with protection against mitochondrial damage in the absence of neuroinflammation.

In vitro studies and studies in animal models of cancer indicate that co-administration of metformin potentiates the anti-cancer effects of radiation and chemotherapeutics, including cisplatin, and cancer stem cell death[25,34]. Meta-analyses of mostly retrospective epidemiological studies in patients treated for diabetes indicate that metformin is associated with decreased incidence of cancer, as compared with other potential treatments or no treatment[53–56]. Unfortunately, cognitive function and neuropathic pain were not included as endpoints in these and other clinical studies on the effect of metformin in cancer patients.

The dosage of metformin that we used in this study was based on earlier studies showing a beneficial effect of metformin on neuropathic pain[30,57,58]. Dosing in mice cannot be directly translated to dosing in humans because of species-related differences in metabolic rate and drug clearance. It should be noted, however, that the dose we used here is in the dose range used for treatment of diabetes in mice [59]. It is important to note that we only studied metformin’s effect when it was started before cisplatin treatment. We showed earlier that metformin did not have any effect on neuropathy when given after completion of cisplatin treatment [30]. We therefore recommend to use a co-administration approach in potential clinical trials.

Even though metformin is considered safe, a future clinical trial should take possible risks into account, including the potential decrease in vitamin B12 levels that has been reported with chronic use of metformin[60]. Patients treated with cisplatin are at risk for nephrotoxicity, and metformin is cleared via the kidneys. Although metformin is safe in patients with diabetes who have impaired renal function[61], close monitoring would be needed in the cancer population.

In conclusion, we demonstrate that metformin protects against cisplatin-induced deficiencies in cognitive function and confirm its protective effects on cisplatin-induced neuropathic pain. These findings are clinically relevant because of increasing evidence that patients treated for cancer with platinum-based compounds frequently develop cognitive impairment and structural abnormalities in the brain. Having a safe, well tolerated, and inexpensive way to protect against these treatment-related harms would be of great benefit to patients and could positively affect adherence to therapy and, as a result, patient outcomes. Our current results urge for a clinical trial on the potential beneficial eff of metformin co-administration on cognitive function and peripheral neuropathy in patients treated with cisplatin.

## Supporting Information

S1 Fig Latency to First Interaction with Novel Object. Mice received three cycles (5 daily injections of 2 .3 mg/kg i.p followed by 5 days without injections) of cisplatin treatment with or without metformin (100 mg/kg i.p.). The latency to the first interaction with novel object was counted. Data were analyzed by two-way ANOVA. (F(1,26) = 0.02, P = 0.88)All data are expressed as mean±SEM. n = 6–8 per group.
(PDF)

S2 Fig Novel Place Recognition Test. Mice received three cycles (5 daily injections of 2 .3 mg/kg i.p followed by 5 days without injections) of cisplatin treatment. Data were analyzed by Independent t-test. (t = 4.096 df = 10; p = 0.002). All data are expressed as mean±SEM. *, p<0.05. n = 10–14 per group.
(PDF)

S1 File Raw Data. (XLSX)

## References

[1] de Moor JS, Mariotto AB, Parry C, Alfano CM, Padgett L, Kent EE, et al Cancer survivors in the United States: prevalence across the survivorship trajectory and implications for care. Cancer Epidemiol Biomarkers Prev. 2013;22: 561–570. 10.1158/1055-9965.EPI-12-1356 23535024 PMC3654837

[2] Ahles TA, Saykin AJ. Candidate mechanisms for chemotherapy-induced cognitive changes. Nat Rev Cancer. 2007;7: 192–201. 17318212 10.1038/nrc2073 PMC3329763

[3] O'Farrell E, MacKenzie J, Collins B. Clearing the air: a review of our current understanding of "chemo fog". Curr Oncol Rep. 2013;15: 260–269. 10.1007/s11912-013-0307-7 23483375

[4] Wefel JS, Schagen SB. Chemotherapy-related cognitive dysfunction. Curr Neurol Neurosci Rep. 2012;12: 267–275. 10.1007/s11910-012-0264-9 22453825

[5] Selamat MH, Loh SY, Mackenzie L, Vardy J. Chemobrain experienced by breast cancer survivors: a meta-ethnography study investigating research and care implications. PLoS One. 2014;9: e108002 10.1371/journal.pone.0108002 25259847 PMC4178068

[6] Moore HC. An Overview of Chemotherapy-Related Cognitive Dysfunction, or 'Chemobrain'. Oncology (Williston Park). 2014;28:797–804 25224480

[7] Vardy J, Tannock I. Cognitive function after chemotherapy in adults with solid tumours. Crit Rev Oncol Hematol. 2007;63: 183–202. 17678745 10.1016/j.critrevonc.2007.06.001

[8] Simo M, Rifa-Ros X, Rodriguez-Fornells A, Bruna J. Chemobrain: a systematic review of structural and functional neuroimaging studies. Neurosci Biobehav Rev. 2013;37: 1311–1321. 10.1016/j.neubiorev.2013.04.015 23660455

[9] Simo M, Root JC, Vaquero L, Ripolles P, Jove J, Ahles T, et al Cognitive and Brain Structural Changes in Lung Cancer Population. J Thorac Oncol. 2015;10(1): 38–45 10.1097/JTO.0000000000000345 25325778 PMC5657249

[10] Lepage C, Smith AM, Moreau J, Barlow-Krelina E, Wallis N, Collin B, et al A prospective study of grey matter and cognitive function alterations in chemotherapy-treated breast cancer patients. Springerplus. 2014;3: 444 10.1186/2193-1801-3-444 25184110 PMC4149682

[11] Janelsins MC, Kesler SR, Ahles TA, Morrow GR.Prevalence, mechanisms, and management of cancer-related cognitive impairment. Int Rev Psychiatry. 2014;26: 102–113. 10.3109/09540261.2013.864260 24716504 PMC4084673

[12] Ahles TA, Saykin AJ, McDonald BC, Furstenberg CT, Cole BF, Hanscom BS, et al Cognitive function in breast cancer patients prior to adjuvant treatment. Breast Cancer Res Treat. 2008;110: 143–152. 17674194 10.1007/s10549-007-9686-5 PMC3114441

[13] Ferguson RJ, McDonald BC, Saykin AJ, Ahles TA. Brain structure and function differences in monozygotic twins: possible effects of breast cancer chemotherapy. J Clin Oncol. 2007;25: 3866–3870. 17761972 10.1200/JCO.2007.10.8639 PMC3329758

[14] Kesler SR, Wefel JS, Hosseini SM, Cheung M, Watson CL, Hoeft F. Default mode network connectivity distinguishes chemotherapy-treated breast cancer survivors from controls. Proc Natl Acad Sci U S A. 2013;110: 11600–11605. 10.1073/pnas.1214551110 23798392 PMC3710809

[15] Winocur G, Wojtowicz JM, Tannock IF. Memory loss in chemotherapy-treated rats is exacerbated in high-interference conditions and related to suppression of hippocampal neurogenesis. Behav Brain Res. 2014;281: 239–244. 10.1016/j.bbr.2014.12.028 25529185

[16] Winocur G, Henkelman M, Wojtowicz JM, Zhang H, Binns MA, Tannock IF. The effects of chemotherapy on cognitive function in a mouse model: a prospective study. Clin Cancer Res. 2012;18: 3112–3121. 10.1158/1078-0432.CCR-12-0060 22467680

[17] Briones TL, Woods J. Dysregulation in myelination mediated by persistent neuroinflammation: possible mechanisms in chemotherapy-related cognitive impairment. Brain Behav Immun. 2014;35: 23–32. 10.1016/j.bbi.2013.07.175 23916895 PMC3858476

[18] Vichaya EG, Chiu GS, Krukowski K, Lacourt TE, Kavelaars A, Dantzer R, et al Mechanisms of chemotherapy-induced behavioral toxicityies. Front Neurosci. 2015;9:131 10.3389/fnins.2015.00131 25954147 PMC4404721

[19] Whitney KA, Lysaker PH, Steiner AR, Hook JN, Estes DD, Hanna NH. Is"chemobrain" a transient state? A prospective pilot study among persons with non-small cell lung cancer. J Support Oncol. 2008;6: 313–321. 18847074

[20] Gan HK, Bernstein LJ, Brown J, Ringash J, Vakilha M, Wang L, et al Cognitive functioning after radiotherapy or chemoradiotherapy for head-and-neck cancer. Int J Radiat Oncol Biol Phys. 2011;81: 126–134. 10.1016/j.ijrobp.2010.05.004 20708851

[21] Fung C, Vaughn DJ. Complications associated with chemotherapy in testicular cancer management. Nat Rev Urol. 2011;8: 213–222. 10.1038/nrurol.2011.26 21403662

[22] Schagen SB, Boogerd W, Muller MJ, Huinink WT, Moonen L, Meinhardt W, et al Cognitive complaints and cognitive impairment following BEP chemotherapy in patients with testicular cancer. Acta Oncol. 2008;47: 63–70. 17934892 10.1080/02841860701518058

[23] Skoogh J, Steineck G, Stierner U, Cavallin-Stahl E, Wilderang U, Wallin A, et al Testicular-cancer survivors experience compromised language following chemotherapy: findings in a Swedish population-based study 3–26 years after treatment. Acta Oncol. 2012;51: 185–197. 10.3109/0284186X.2011.602113 21851186

[24] Yang JC, Hirsh V, Schuler M, Yamamoto N, O'Byrne KJ, Mok TS, et al Symptom control and quality of life in LUX-Lung 3: a phase III study of afatinib or cisplatin/pemetrexed in patients with advanced lung adenocarcinoma with EGFR mutations. J Clin Oncol. 2013;31: 3342–3350. 10.1200/JCO.2012.46.1764 23816967

[25] Bost F, Sahra IB, Le Marchand-Brustel Y, Tanti JF. (2012) Metformin and cancer therapy. Curr Opin Oncol 24: 103–108. 10.1097/CCO.0b013e32834d8155 22123231

[26] Hinduja S, Kraus KS, Manohar S, Salvi RJ. D-Methionine Protects Against Cisplatin-Induced Neurotoxicity in the Hippocampus of the Adult Rat. Neurotox Res. 2015;27(3):199–204 10.1007/s12640-014-9503-y 25488710 PMC4376112

[27] Manohar S, Jamesdaniel S, Salvi R. Cisplatin inhibits hippocampal cell proliferation and alters the expression of apoptotic genes. Neurotox Res. 2014;25: 369–380. 10.1007/s12640-013-9443-y 24277158 PMC3972319

[28] Song TY Chen CL, Liao JW, Ou HC, Tsai MS. Ergothioneine protects against neuronal injury induced by cisplatin both in vitro and in vivo. Food Chem Toxicol. 2010;48(12):3492–3499 10.1016/j.fct.2010.09.030 20932872

[29] Giridharan VV, Thandavarayan RA, Bhilwade HN, KoKM, Watanabe K, Konishi T. Schisandrin B, attenuates cisplatin-induced oxidative stress, genotoxicity and neurotoxicity through modulating NF-kappaB pathway in mice. Free Radic Res. 2012;46:50–60 10.3109/10715762.2011.638291 22059853

[30] Mao-Ying QL, Kavelaars A, Krukowski K, Huo XJ, Zhou W, Price TJ, et al The anti-diabetic drug metformin protects against chemotherapy-induced peripheral neuropathy in a mouse model. PLoS One, 2014;9: e100701 10.1371/journal.pone.0100701 24955774 PMC4067328

[31] Labuzek K, Suchy D, Gabryel B, Bielecka A, Liber S, Okopien B. Quantification of metformin by the HPLC method in brain regions, cerebrospinal fluid and plasma of rats treated with lipopolysaccharide. Pharmacol Rep. 2010;62: 956–965. 21098880 10.1016/s1734-1140(10)70357-1

[32] Emami Riedmaier A, Fisel P, Nies AT, Schaeffeler E, Schwab M. Metformin and cancer: from the old medicine cabinet to pharmacological pitfalls and prospects. Trends Pharmacol Sci. 2013;34(2): 126–135 10.1016/j.tips.2012.11.005 23277337

[33] Le Merrer J, Rezai X, Scherrer G, Becker JAJ, Kieffer B. (2013) Impaired hippocampus dependent and facilitated striatum-dependent behaviors in mice lacking the delta opioid receptor. Neuropsychopharmacology 38(6): 1050–1059 10.1038/npp.2013.1 23303070 PMC3629400

[34] Engelmann M, Hädicke, Noack J. Testing declarative memory in laboratory rats and mice using the nonconditioned social discrimination procedure. Nature Protocols. 2011;6:1152–1162 10.1038/nprot.2011.353 21799485

[35] Wang H, Heijnen CJ, van Velthoven CTJ, Willemen HLDM, Ishikawa Y, Zhang X, et al Balancing GRK2 and EPAC1 levels prevents and relieves chronic pain. J Clin Invest. 2013;123: 5023–5034. 10.1172/JCI66241 24231349 PMC3859388

[36] Donega V, van Velthoven CTJ, Nijboer CH, van Bel F, Kas MJH, Kavelaars A, et al Intranasal mesenchymal stem cell treatment for neonatal brain damage: long-term cognitive and sensorimotor improvement. PLoS One. 2013;8(1): e51253 10.1371/journal.pone.0051253 23300948 PMC3536775

[37] Bevins RA, Besheer J. Object recognition in rats and mice: a one-trial non-matching-to-sample learning task to study 'recognition memory'. Nat Protoc. 2006;1: 1306–1311. 17406415 10.1038/nprot.2006.205

[38] Guterstam A, Björnsdoter M, Gentile G, Ehrsson HH. Posterior cingulate cortex integrates the senses of self-location and body ownership. Current Biology. 20115;25(11): 1416–1425 10.1016/j.cub.2015.03.059 25936550

[39] Miller AM, Vedder L, Law LM, Smith DM. Cues, context, and long-term memory: the role of the retrosplenital cortex in spatial cognition. Frontiers in Human Neuroscience. 2014;8: 586 10.3389/fnhum.2014.00586 25140141 PMC4122222

[40] Yoon SY, Robinson CR, Zhang H, Dougherty PM. Spinal astrocyte gap junctions contribute to oxaliplatin-induced mechanical hypersensitivity. J Pain. 2013;14: 205–214. 10.1016/j.jpain.2012.11.002 23374942 PMC3564051

[41] Di Cesare Mannelli L, Pacini A, Micheli L, Tani A, Zanardelli M, Ghelardini C. Glial role in oxaliplatin-induced neuropathic pain. Exp Neurol. 2014;261: 22–33. 10.1016/j.expneurol.2014.06.016 24967684

[42] Robinson CR, Zhang H, Dougherty PM. Astrocytes, but not microglia, are activated in oxaliplatin and bortezomib-induced peripheral neuropathy in the rat. Neuroscience. 2014;274: 308–317. 10.1016/j.neuroscience.2014.05.051 24905437 PMC4099296

[43] Bourne JN, Harris KM. Balancing structure and function at hippocampal dendritic spines. Annu Rev Neurosci. 2008;31: 47–67. 10.1146/annurev.neuro.31.060407.125646 18284372 PMC2561948

[44] Lee KF, Soares C, Beique JC. Examining form and function of dendritic spines. Neural Plast. 2012;2012: 704103 10.1155/2012/704103 22577585 PMC3345238

[45] Yuste R. Dendritic spines and distributed circuits. Neuron. 2011;71: 772–781. 10.1016/j.neuron.2011.07.024 21903072 PMC4071954

[46] Ultanir SK, Kim JE, Hall BJ, Deerinck T, Ellisman M, Ghosh A. Regulation of spine morphology and spine density by NMDA receptor signaling in vivo. Proc Natl Acad Sci U S A. 2007;104: 19553–19558. 18048342 10.1073/pnas.0704031104 PMC2148327

[47] Jiang T, Yu JT, Zhu XC, Wang HF, Tan MS, Cao L, et al Acute metformin preconditioning confers neuroprotection against focal cerebral ischaemia by pre-activation of AMPK-dependent autophagy. Br J Pharmacol. 2014;171: 3146–3157. 10.1111/bph.12655 24611741 PMC4080970

[48] Li J, Benashski SE, Venna VR, McCullough LD. Effects of metformin in experimental stroke. Stroke. 2010;41: 2645–2652. 10.1161/STROKEAHA.110.589697 20847317 PMC2994809

[49] Liu Y, Tang G, Li Y, Wang Y, Chen X, Gu X, et al Metformin attenuates blood-brain barrier disruption in mice following middle cerebral artery occlusion. J Neuroinflammation. 2014;11: 177 10.1186/s12974-014-0177-4 25315906 PMC4201919

[50] Park HJ, Stokes JA, Pirie E, Skahen J, Shtaerman Y, Yaksh TL. Persistent Hyperalgesia in the cisplatin-treated mouse as defined by threshold measures, the conditioned place preference paradigm, and changes in dorsal root ganglia activated transcription factor3: the effects of gabapentin, ketorolac, and etanercept. Anesth Analg. 2013;116(1):224–231 10.1213/ANE.0b013e31826e1007 23223118 PMC3530135

[51] Valero T. Mitochondrial biogenesis: pharmacological approaches. Curr Pharm Des. 2014;20: 5507–5509. 24606795 10.2174/138161282035140911142118

[52] Chung MM, Chen YL, Pei D, Cheng YC, Sun B, Nicol CJ, et al The neuroprotective role of metformin in advanced glycation end product treated human neural stem cells is AMPK-dependent. Biochim Biophys Acta. 2015;1852: 720–731. 10.1016/j.bbadis.2015.01.006 25595658

[53] Decensi A, Puntoni M, Goodwin P, Cazzaniga M, Gennari A, Bonanni B, et al Metformin and cancer risk in diabetic patients: a systematic review and meta-analysis. Cancer Prev Res (Phila). 2010;3: 1451–1461. 20947488 10.1158/1940-6207.CAPR-10-0157

[54] Wu L, Zhu J, Prokop LJ, Murad MH. Pharmacologic Therapy of Diabetes and Overall Cancer Risk and Mortality: A Meta-Analysis of 265 Studies. Sci Rep. 2015;5: 10147 10.1038/srep10147 26076034 PMC4467243

[55] Franciosi M, Lucisano G, Lapice E, Strippoli GF, Pellegrini F, Nicolucci A. Metformin therapy and risk of cancer in patients with type 2 diabetes: systematic review. PLoS One. 2013;8: e71583 10.1371/journal.pone.0071583 23936520 PMC3732236

[56] Noto H, Goto A, Tsujimoto T, Noda M. Cancer risk in diabetic patients treated with metformin: a systematic review and meta-analysis. PLoS One. 2012;7: e33411 10.1371/journal.pone.0033411 22448244 PMC3308971

[57] Melemedjian OK, Yassine HN, Shy A, Price TJ. Proteomic and functional annotation analysis of injured peripheral nerves reveals ApoE as a protein upregulated by injury that is modulated by metformin treatment. Mol Pain. 2013;9: 14 10.1186/1744-8069-9-14 23531341 PMC3623807

[58] Melemedjian OK, Asiedu MN, Tillu DV, Sanoja R, Yan J, Lark A, et al Targeting adenosine monophosphate-activated protein kinase (AMPK) in preclinical models reveals a potential mechanism for the treatment of neuropathic pain. Mol Pain. 2011;7: 70 10.1186/1744-8069-7-70 21936900 PMC3186752

[59] Yoon SH, Han EJ, Sung JH, Chung SH. Anti-diabetic effects of compound K versus metformin versus compound K-metformin combination therapy in diabetic db/db mice. Biol Pharm Bull. 2007;30: 2196–2200. 17978500 10.1248/bpb.30.2196

[60] Wile DJ, Toth C. Association of metformin, elevated homocysteine, and methylmalonic acid levels and clinically worsened diabetic peripheral neuropathy. Diabetes Care. 2010;33: 156–161. 10.2337/dc09-0606 19846797 PMC2797962

[61] Inzucchi SE, Lipska KJ, Mayo H, Bailey CJ, McGuire DK. Metformin in patients with type 2 diabetes and kidney disease: a systematic review. JAMA. 2014;312: 2668–2675. 10.1001/jama.2014.15298 25536258 PMC4427053

