# Supplementary figures and images for: Metformin Prevents Cisplatin-Induced Cognitive Impairment and Brain Damage in Mice
Source: PLoS One. 2016 Mar 28;11(3):e0151890. doi: 10.1371/journal.pone.0151890 (PMC4809545; doi:10.1371/journal.pone.0151890)

### Latency to First Interaction with Novel Object

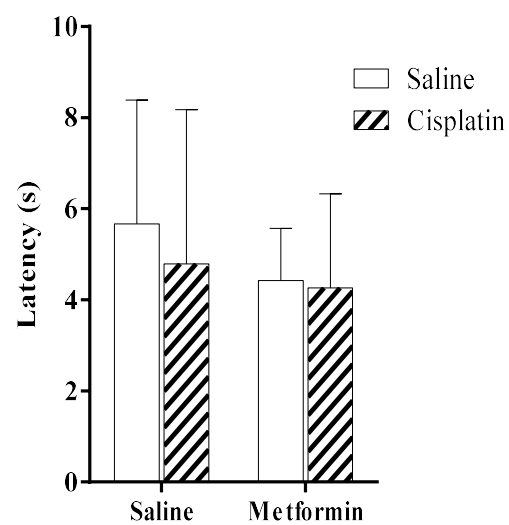

Supplement: S1 Fig — Mice received three cycles (5 daily injections of 2 .3 mg/kg i.p followed by 5 days without injections) of cisplatin treatment with or without metformin (100 mg/kg i.p.). The latency to the first interaction with novel object was counted. Data were analyzed by two-way ANOVA. (F(1,26) = 0.02, P = 0.88)All data are expressed as mean±SEM. n = 6–8 per group. (PDF) [file pone.0151890.s001.pdf]

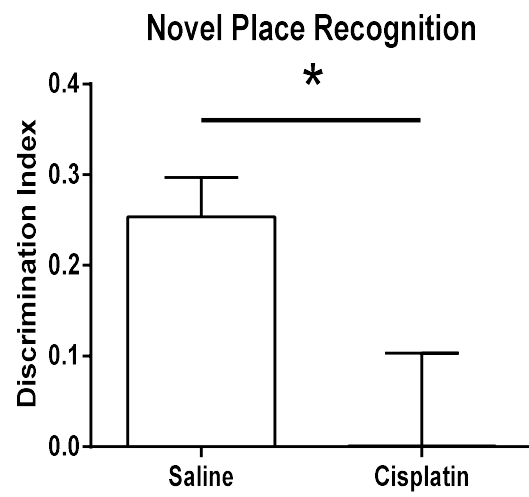

Supplement: S2 Fig — Mice received three cycles (5 daily injections of 2 .3 mg/kg i.p followed by 5 days without injections) of cisplatin treatment. Data were analyzed by Independent t-test. (t = 4.096 df = 10; p = 0.002). All data are expressed as mean±SEM. *, p<0.05. n = 10–14 per group. (PDF) [file pone.0151890.s002.pdf]
